# DC vs AC Electrokinetics-Driven Nanoplasmonic Raman Monitoring of Charged Analyte Molecules in Ionic Solutions

**DOI:** 10.1021/acs.jpcc.4c04485

**Published:** 2024-08-31

**Authors:** Chuan Xiao, Xin Wang, Yuming Zhao, Hongwei Zhang, Junyeob Song, Peter Vikesland, Rui Qiao, Wei Zhou

**Affiliations:** † Department of Electrical and Computer Engineering, Virginia Tech, Blacksburg, Virginia 24061, United States; ‡ Department of Mechanical Engineering, Virginia Tech, Blacksburg, Virginia 24061, United States; § Department of Civil and Environmental Engineering, Virginia Tech, Blacksburg, Virginia 24061, United States

## Abstract

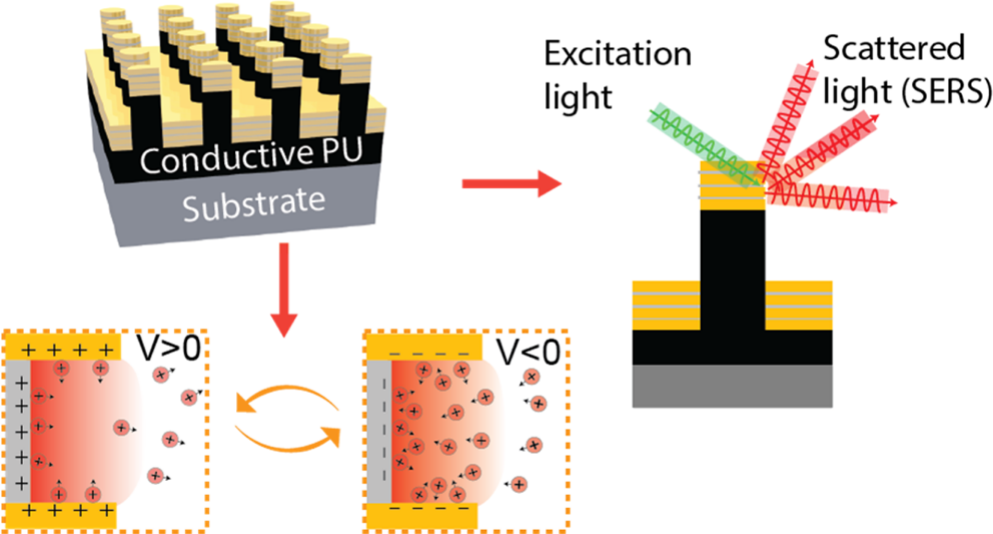

Electrokinetic surface-enhanced Raman spectroscopy (EK-SERS) is an emerging high-order analytical technique that combines the plasmonic sensitivity of SERS with the electrode interfacial molecular control of electrokinetics. However, previous EK-SERS works primarily focused on non-Faradaic direct current (DC) operation, limiting the understanding of the underlying mechanisms. Additionally, developing reliable EK-SERS devices with electrically connected plasmonic hotspots remains challenging for achieving high sensitivity and reproducibility in EK-SERS measurements. In this study, we investigated the use of two-tier nanolaminate nano-optoelectrode arrays (NL-NOEAs) for DC and alternating current (AC) EK-SERS measurements of charged analyte molecules in ionic solutions. The NL-NOEAs consist of Au/Ag/Au multilayered plasmonic nanostructures on conductive nanocomposite nanopillar arrays (NC-NPAs). We demonstrate that the NL-NOEAs exhibit high SERS enhancement factors (EFs) of ∼10^6^ and can be used to modulate the concentration and orientation of Rhodamine 6G (R6G) molecules at the electrode surface by applying DC and AC voltages. We also performed numerical simulations to investigate the ion and R6G dynamics near the electrode surface under DC and AC voltage modulation. Our results show that AC EK-SERS can provide additional insights into the dynamics of molecular transport and adsorption processes compared to DC EK-SERS. This study demonstrates the potential of NL-NOEAs for developing high-performance EK-SERS sensors for a wide range of applications.

## Introduction

Electrochemical surface-enhanced Raman spectroscopy (EC-SERS) has emerged as a powerful high-order spectroelectrochemistry tool by combining the plasmonic hotspot vibrational fingerprinting sensitivity of SERS with electrochemistry interfacial molecular control offered by electrode.^1,2^ This synergistic approach has enabled researchers to delve into the intricate molecular interactions at electrode surfaces, paving the way for advancements in fields like electrocatalysis, biosensing, and materials characterization.^3−5^ The core principle of SERS lies in the dramatic enhancement of Raman scattering signals when molecules are adsorbed onto nanostructured metal surfaces.^6,7^ This enhancement is primarily attributed to the localized surface plasmon resonance (LSPR) effect, where incident light excites collective oscillations of electrons in the metal nanostructures, generating intensified electromagnetic fields that significantly increase both the excitation rate and emission efficiency in Raman scattering processes.^8,9^ By integrating electrochemistry, EC-SERS allows for precise control over the electrode potential, facilitating the manipulation of molecular adsorption, desorption, orientation, and even triggering interfacial electrochemical reactions.^3−5^ This added EC control to SERS measurements can potentially introduce high-order analytical advantage to significantly improve analytical selectivity, sensitivity, and calibration.^10−12^

As a subtype of EC-SERS, previous research on non-Faradaic electrokinetic (EK) SERS has mainly focused on modulating the electrode potential with mild direct current (DC) voltage to manipulate the concentration and orientation of molecules at the electrode surface without inducing interfacial electrochemical reaction. For instance, EK-SERS can be used to probe electric fields in the diffuse double layer^13−15^ and even map molecule orientation changes at hotspots.^16−18^ Other researchers have utilized electrokinetic phenomena, such as electrophoresis and dielectrophoresis, to enrich molecules at SERS hotspots for enhanced sensitivity,^19−25^ and to deplete adsorbed molecules for SERS substrate regeneration.^26^ In addition, EK-SERS has the potential to develop high-order analytical methods for multianalyte detection.^27^ While DC-based EK-SERS has provided valuable insights, the dynamic nature of molecular transport and adsorption processes necessitates the exploration of alternative modulation techniques. Most previous non-Faradaic EK-SERS works primarily focused on DC operation, limiting the understanding of the underlying interfacial molecular dynamics and transport mechanisms. The use of alternating current (AC) voltage modulation in EK-SERS is a promising approach to gain further insights into these dynamic processes.^20^ AC EK-SERS allows for the investigation of molecular behavior under nonequilibrium conditions, revealing information about adsorption kinetics, molecular reorientation, and transport phenomena that are not accessible with DC EK-SERS. However, the lack of systematic studies on AC EK-SERS, especially with quantitative analysis and theoretical modeling, leaves a significant knowledge gap in the field.

Additionally, the development of effective SERS substrates with electrically connected plasmonic hotspots has been a persistent challenge for achieving high sensitivity and reproducibility in EC-SERS measurements. While various EC-SERS and EK-SERS substrate types have been explored, including nanostructured metal electrodes,^13−15,17,20−22,25−28^ nanoparticle aggregates on electrodes,^23,24^ nanoparticles on a mirror (NPoM),^29,30^ and shell-isolated nanoparticle-enhanced Raman spectroscopy (SHINERS) substrates,^31,32^ limitations persist in terms of hotspot sensitivity, uniformity, mechanical stability, and manufacturing scalability. For instance, roughened metal electrodes, although easy to fabricate over a large area, are less reproducible and have poor SERS sensitivity. Nanoparticle aggregates on electrodes, NPoM, and SHINERS substrates, though highly sensitive, are difficult to fabricate uniformly over large areas and often lack mechanical robustness. These limitations underscore the need for innovative approaches to develop scalable SERS substrates with uniform and stable hotspots for practical EC-SERS applications.

In this study, we present a novel approach to EK-SERS by utilizing two-tier nanolaminate nano-optoelectrode arrays (NL-NOEAs) for both DC and AC EK-SERS measurements of charged analyte molecules in ionic solutions. The NL-NOEAs consist of Au/Ag/Au multilayered plasmonic nanostructures on conductive nanocomposite nanopillar arrays (NC-NPAs), offering high SERS enhancement factors (EFs) of ∼10^6^. We demonstrate the capability of NL-NOEAs to modulate the concentration and orientation of Rhodamine 6G (R6G) molecules at the electrode surface by applying DC and AC voltages. Furthermore, we performed modeling and numerical simulations to investigate the ion and R6G dynamics near the electrode surface under DC and AC voltage modulation, revealing that AC EK-SERS can provide additional insights into the dynamics of molecular transport and adsorption processes compared to DC EK-SERS. This study highlights the potential of NL-NOEAs as a versatile platform for high-order DC and AC EK-SERS analysis, offering a new avenue for the development of advanced sensing techniques with enhanced sensitivity, selectivity, and dynamic information for a wide range of applications.

## Methods

### Fabrication of Conductive NL-NOEAs

The fabrication process involved several crucial steps to ensure optimal performance. (1) A nanowell composite poly(dimethylsiloxane) (PDMS) stamp, consisting of a hard and soft PDMS mixture, was fabricated from a nanopillar-structured silicon wafer (120 nm diameter, 150 nm height, 400 nm period). (2) UV-curable polyurethane (PU) (NOA83H) was nanoimprinted with the PDMS stamp to create nanopillar arrays on a polyester film, followed by ultraviolet (UV) and thermal curing. (3) The PU nanopillar array (NPA) was treated with tridecafluoro-1,1,2,2-tetrahydrooctyl-1-tri (TFCOS) in a vacuum desiccator for 30 min, providing a hydrophobic surface. (4) The PU NPA was molded with photoinitiator-added perfluoropolyether (PFPE) to generate nanowell arrays on a polyester film, followed by UV and thermal curing. (5) A 20 wt % MWCNT/NOA83H composite resist was imprinted with the PFPE nanowell stamp to create nanopillar nanoelectrode arrays (NC-NPAs) on a Si wafer substrate, followed by UV and thermal curing. (6) Electron-beam deposition was utilized to deposit alternating layers of Au and Ag (total of 7 layers, 126 nm thickness) on the NC-NPAs, forming multilayered NL-NOEAs. The Au layer was 35 nm thick, while the Ag layers had sequential thicknesses of 6, 8, and 12 nm from bottom to top. Notably, 1 nm of Cr and 0.5 nm of Ti were deposited as adhesion layers between the NC-NPAs and the first layer of Au, and between the Au and Ag layers, respectively.

### SERS Measurement

To assess SERS performance on NL-NOEAs samples, benzenethiol (BZT) (CAS: 108–98–5, Sigma-Aldrich) as a probe molecule. A self-assembled monolayer (SAM) of BZT was formed on the substrate by incubating it in a 1 mM BZT ethanol solution for 18 h, followed by rinsing with ethanol and drying with a gentle nitrogen stream. Raman measurements were performed using a confocal Raman microscopy system (alpha300 RS+, WITec), which allowed the simultaneous introduction of the incident laser and collection of backscattered signals via a collimator, beam splitter, and long-pass filter designed for 785 nm. The laser power was set at 2 mW, and the integration time was 0.5 s per pixel, ensuring accurate and precise signal detection from the BZT-modified substrates. To minimize the impact of fluorescence background in the R6G measurements, we employed shape background subtraction during data processing using the WITec software. This technique fits a smooth curve to the baseline of the spectrum, effectively removing the broad fluorescence background while preserving the sharp Raman peaks.

### Optical Measurement

Reflectance spectra of NL-NOEAs samples in water (refractive index, 1.33) were obtained using a UV-visible-near infrared (UV–vis-NIR) spectrophotometer (Cary 5000, Varian).

### SERS Enhancement Factor (SERS EF) Assessment

The SERS EF was calculated using the equation: SERS EF = (*I*_SERS_/*I*_Raman_) × (*N*_Raman_/*N*_SERS_),^33^ where *I*_SERS_, *I*_Raman_, *N*_SERS_, and *N*_Raman_ represent the measured SERS intensity, neat BZT Raman intensity, and the number of BZT molecules contributing to SERS and neat Raman intensities, respectively. The BZT peak at 1094 cm^–1^ (in-plane C–C–C ring breathing mode with C–S stretching mode) was used for *I*_Raman_, while the shifted SERS peak of BZT at 1078 cm^–1^, due to the absorption onto the metallic surface, was used for *I*_SERS_,. *N*_SERS_ was calculated using the equation *N*_SERS_ = SA × ρ_SERS_, where SA denotes the surface area of SERS substrates (i.e., NL-NOEAs samples) contributing to the Raman signal enhancement, and ρ_SERS_ refers to the interfacial packing density of BZT on the Au surface (6.8 × 10^14^ molecules/cm^2^). *N*_Raman_ was determined with the equation *N*_Raman_ = *A*_deff_*× ρ*_BZT_, where *A* is the area of the focused illumination, *d*_eff_ is the effective focal depth of the beam spot, and ρ_BZT_ is the volumetric density of neat BZT molecules (5.9 × 10^21^ molecules/cm^3^). The effective focal depth, *d*_eff_, was measured using a silicon wafer by varying the distance between the wafer and the objective lens.

### Electrochemical Measurement

Electrochemical impedance spectroscopy (EIS) was performed using a potentiostat (SP-200, Biologic Science Instrument) with the working electrode voltage (*V*_WE_) set to zero vs open circuit and a sinus amplitude of 10 mV. The equivalent circuit model assumes a current-less situation (*I*_DC_ = 0) with no applied direct current or voltage. An Ag/AgCl electrode saturated in KCl served as the reference electrode, and a platinum wire functioned as the counter electrode in a custom electrochemical cell (EC-cell) mounted on the sample. Phosphate buffer saline (PBS) was utilized as the electrolyte solution, composed of 137 mM NaCl, 2.7 mM KCl, 10 mM Na_2_HPO_4_, and 1.8 mM KH_2_PO_4_ for standard 1× PBS from commercial suppliers. Approximately 0.55 mL of 1× PBS was added to the EC-cell, and measurements were conducted at room temperature. The SP-200 potentiostat was employed to carry out EIS and cyclic voltammetry (CV) in the range of −0.6 to 0.4 V with 50 mV/s increments. CV measurements were cycled multiple times until the electrochemical system reached a steady state, yielding consistent CV outputs before initiating the measurements.

### EK-SERS Measurement

For conducting EK-SERS measurements, a custom EC cell was integrated into the scanning stage of the confocal Raman microscopy system while electrically connected to the potentiostat. EK-SERS measurements were performed on 10^–5^ M R6G (CAS: 989–38–8, Sigma-Aldrich) in 1× PBS. In DC EK-SERS measurements, the desired DC voltage input was initiated ∼10 s before the SERS measurement and lasted for 130 s. Each DC EK-SERS measurement was acquired by scanning a 10 μm × 10 μm area using a 10× objective lens (numerical aperture, NA: 0.9) with 10 × 10 pixels under the excitation of a 785 nm diode laser (2 mW power) and an integration time of 1 s per pixel. For AC EK-SERS measurements, a −0.6 to 0.4 V CV input was applied at a scanning speed of 50 mV/s. Each AC EK-SERS measurement was acquired by scanning a 10 μm × 20 μm area using a 10× objective lens with 10 × 20 pixels, maintaining the same excitation and integration time as in DC EK-SERS measurements.

### Molecule Dynamic Simulation

The simulation system used to study the adsorption of R6g molecules on gold electrodes was built in our previous work.^34,35^ This system features two parallel gold electrodes, two R6g molecules, water molecules, Na^+^ and Cl^–^ ions. Two R6g molecules are placed within 1 nm from the upper and lower gold surfaces, respectively. The distance between the gold surfaces that are exposed to the NaCl solution is 5 nm, which is wide enough to generate bulk-like water in the middle of the system. The concentration of NaCl in the middle of the system is ∼0.27 M. The system is periodical in *xy*- directions. The simulation box measures 4.894 × 4.894 × 20 nm^3^ and each gold substrate measures 4.894 × 4.894 × 0.816 nm^3^. Two vacuum spaces with a height of 6.684 nm are placed above the top electrode and below the bottom electrode. Starting from a random configuration, an energy minimization step is conducted using the steepest descent algorithm until the maximal force is below 1000 kJ mol^–1^ nm^–1^. Next, an NVT equilibrium run of 105 ns is conducted. Statistics are taken from the last 5 ns.

The gold surface exposed to the solution is (001). A small atomic charge of −0.0625*e*, or +0.04167*e* is placed on each gold atom in contact with the NaCl solution to mimic the desired voltages of gold electrodes of −0.6 and +0.4 V. Simulations are performed using the GROMACS 2020.3 code^36^ in the NVT ensemble. The force field parameters for gold atoms were taken from previous work.^35,37^ The TIP3P model is adopted for the water molecules.^38^ The force field parameters for R6G are taken from the CHARMM27 force fields.^35^ Na^+^ and Cl^–^ ions are described using the force fields by Joung and Cheatham.^39^ The gold atoms are fixed. The temperature of all mobile molecules is maintained at 300 K using a velocity rescaling thermostat with a time constant of 0.1 ps. A time step of 2 fs is used. The particle mesh Ewald (PME) method calculates the electrostatic interactions, and the fast Fourier transform (FFT) spacing is taken as 0.12 nm. The slab correction is applied to remove the periodicity in the *z*-direction. A cutoff length of 1.2 nm is employed for nonelectrostatic interactions and the real-space part of the PME calculation. The force switch is adopted as a Lennard-Jones force modifier, and the switching distance is 1.0 nm.

### Theoretical Analysis of SERS Peak Ratio with EK Modulation

To enhance our comprehension of the EK process, we formulated the following equation for EK-SERS signal intensity, drawing upon the established SERS equation^40^
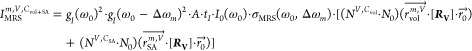
1In this equation, “SA” denotes terms associated with R6G molecules specifically adsorbed to the metal surface within the plasmonic hotspot. “Vol” denotes terms associated with combined volumetric contributions of freely moving R6G molecules in the bulk solution and those nonspecifically adsorbed within the plasmonic hotspot. *g*_*I*_ is the average field-enhancement factor at excitation frequency (ω_0_) or Raman scattering frequency (ω_0_ – Δω_*m*_) within the plasmonic hotspot. A is the metal surface area contributing to the hotspot, and *t*_*I*_ is the average field depth within the plasmonic hotspot. *I*_0_ is the intensity of incident laser. σ_MRS_(ω_0_, Δω_*m*_) is the effective Raman scattering cross-section for vibrational mode (m). *N*^*V*,*C*_vol_^ and *N*^*V*,*C*_SA_^ represent the relative volumetric and specifically adsorbed molecule concentrations in the hotspot, respectively, under a specific electrode voltage (V). *N*_0_ is the max concentration of R6G in hotspot at a specific bulk concentration.  is the local electric field direction vector of plasmonic modes at hotspots.  and  are the relative orientation vectors between the transition dipole moment of mode m and a reference orientation, for volumetric and specifically adsorbed molecules, respectively, at voltage V. [***R***_**V**_] is the effective matrix coefficient relating the reference molecular orientation to the local electric field direction at the hotspot under voltage V.

For Figures 4B and 6C,D, we derive the following equation
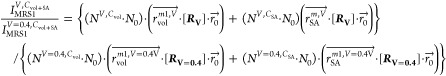
2Assuming all positively charged volumetric molecules are repelled from the hotspot at *V* = 0.4 V (i.e., *N*^*V*=0.4,*C*_vol_^ = 0), the equation simplifies to
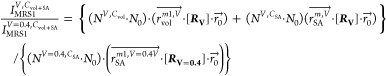
3In the positive range (0.1–0.4 V), *N*^*V*,*C*_vol_^ decreases to 0. When *N*^*V*,*C*_vol_^ = 0 (all volumetric molecules repelled from the hotspot), the equation contains only the orientation info of SA molecules
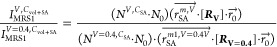
4The pattern of these intensity ratios for multiple peaks at a given voltage, relative to 0.4 V, can act as a unique fingerprint for molecules like R6G.

For Figures 4C and 6E,F, we examine the ratio of peak intensities for mode *m*_2_ to that of the 1312 cm^–1^ peak at the same voltage
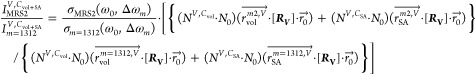
5This ratio depends on both the Raman cross sections and molecular orientations. Assuming the ratio of Raman cross sections for the two modes is constant , we obtain information primarily related to molecular orientation. In the positive voltage range (0.1 to 0.4 V), *N*^*V*,*C*_vol_^ is relatively small compared to *N*^*V*,*C*_SA_^, and the equation simplifies further
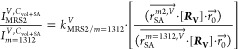
6In the negative range (−0.1 to −0.6 V), the intensity ratio increase is limited, suggesting SA molecules cannot be neglected in the analysis. This behavior can also serve as a distinguishing characteristic for different molecules.

### Simulation Studies

As proof of concept to understand the R6G behaviors above, the shape of the electrode is approximately taken as a sphere (radius: *R*_0_ = 1 μm). The solution surrounding the electrode is treated as the domain of interest and is treated as a shell (the radius of the outer layer: *R* = 10 μm). The position of the outer layer is chosen to be far from the electrode so that the solution concentrations remain at bulk values. The solution consists of 167 mM NaCl and 10 μM R6G. The nonelectrostatic interfacial force corresponding to the R6G adsorption is mimicked as an external attractive force to R6G in the region near the electrode. The voltage applied on the electrode is reproduced by the boundary condition. The governing equations for the spatiotemporal evolution of ion concentrations under spherical coordinates are
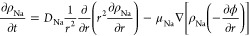
7
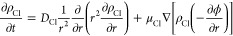
8
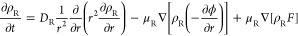
9
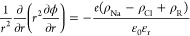
10where ρ_Na_, ρ_Cl_, and ρ_R_ are the concentration of the Na^+^, Cl^–,^ and R6G^+^ ions in the solution, respectively. μ_*Na*_, μ_*Cl*,_ and μ_*Cl*_ are the mobility of the Na^+^ and Cl^–^ ions. The diffusivity and mobility are related by the Einstein relation *D =* μ*k*_B_*T*/*e* where *e* is the elementary charge. ϕ is voltage, and ε_r_ is the relative dielectric permittivity of the solution. *F* is the attractive force to R6G exclusively existing within 1 μm from the electrode to mimic the adsorption. The equation set is closed by the following initial and boundary conditions
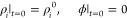
11
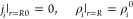
12
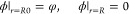
13where ρ_*i*_^0^ is the initial ion concentration of ions that equal to the bulk values. *j*_*i*_ = 0 corresponds to the no flux boundary condition of ions at the electrode–solution interface. Depending on the type of voltage on electrode, φ could be either constant or periodic.

## Results and Discussion

**Figure 1:**
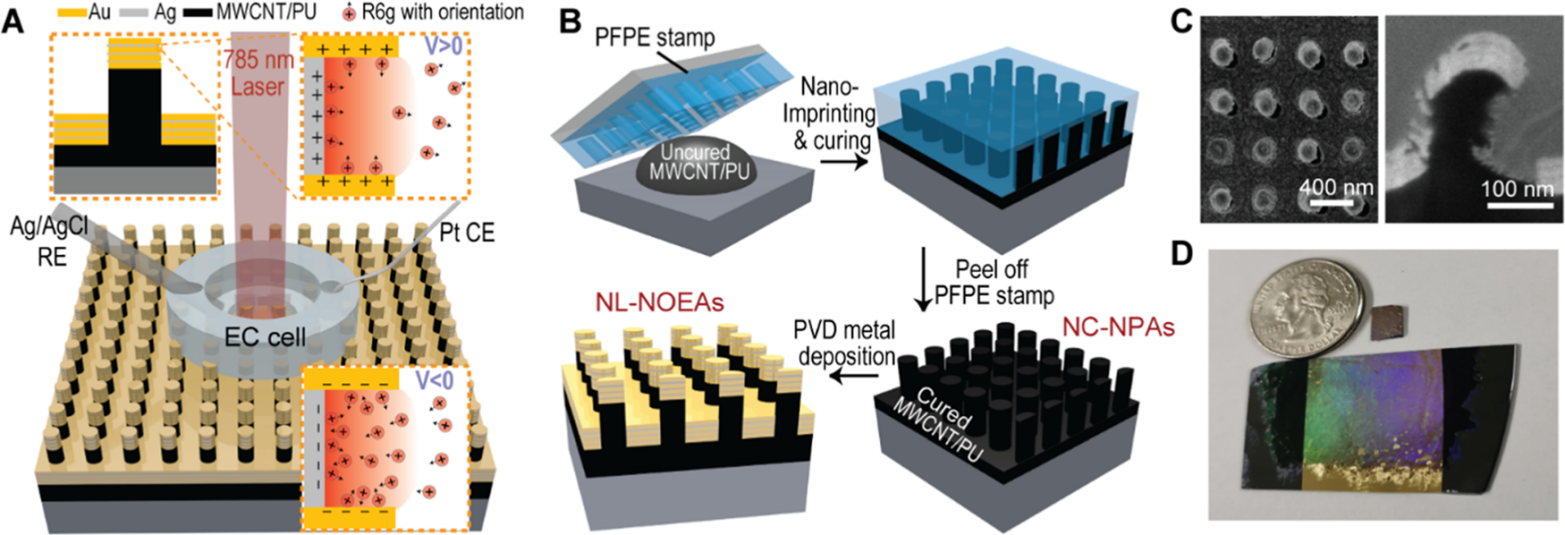
Two-tier nanolaminate nano-optoelectrode arrays (NL-NOEAs) for electrokinetic surface-enhanced Raman spectroscopy (EK-SERS) measurements of charged analyte molecules in ionic solutions. (A) Schematic diagram illustrating EK-SERS detection of Rhodamine 6G (R6G) using NL-NOEAs under positive and negative voltage biases. The NL-NOEAs comprise electrically connected Au/Ag/Au multilayered plasmonic hotspots integrated onto multiwalled carbon nanotube/polyurethane (MWCNT/PU)-based conductive nanocomposite nanopillar arrays (NC-NPAs). (B) Schematic illustration of the NL-NOEAs fabrication process. (C) Top-down (left) and cross-sectional (right) scanning electron microscopy (SEM) images of the NL-NOEA substrate, with focused ion beam (FIB) milling used for cross-section preparation. (D) Photograph of NL-NOEAs on a silicon wafer alongside a quarter coin and a sectioned sample for size comparison.

The fabrication of the two-tier NL-NOEAs utilized a scalable nanoimprinting process (Figure 1B). NC-NPAs were produced using a combination of UV and thermal curing nanoimprinting with a 20 wt % MWCNT/NOA83H nanocomposite.^41^ Alternating layers of Au (25 nm) and Ag (6, 8, and 12 nm) were then deposited onto the NC-NPAs via electron-beam evaporation. Our previous work^42^ has shown that alternating gold (Au) and dielectric materials can create plasmonic nanogap nanocavities, resulting in exceptionally high SERS enhancement factors (EFs) exceeding 10^7^. However, to maintain the device’s electrical conductivity essential for electrokinetic modulation, we replaced the dielectric layer with silver (Ag). While this may reduce the SERS EF (∼10^6^), the resulting Au/Ag/Au multilayered nanolaminate structure ensures efficient electron transport throughout the device, enabling its function as an electrode for EK-SERS. Characterization by top-view and cross-sectional scanning electron microscopy (SEM) revealed a periodicity of ≈400 nm, nanopillar diameters of ≈100 nm, and nanopillar heights of ≈250 nm for NL-NOEAs (Figure 1C). The deviation from a perfect cylindrical nanopillar shape is attributed to the high viscosity of the uncured MWCNT/NOA83H nanocomposite resist, which can influence the nanoimprinting process. The scalable NL-NOEA samples can reach up to 4 × 4 cm^2^ in area (Figure 1D), serving as the working electrode (WE) for interfacial impedance characterization and EK-SERS measurements.

**Figure 2:**
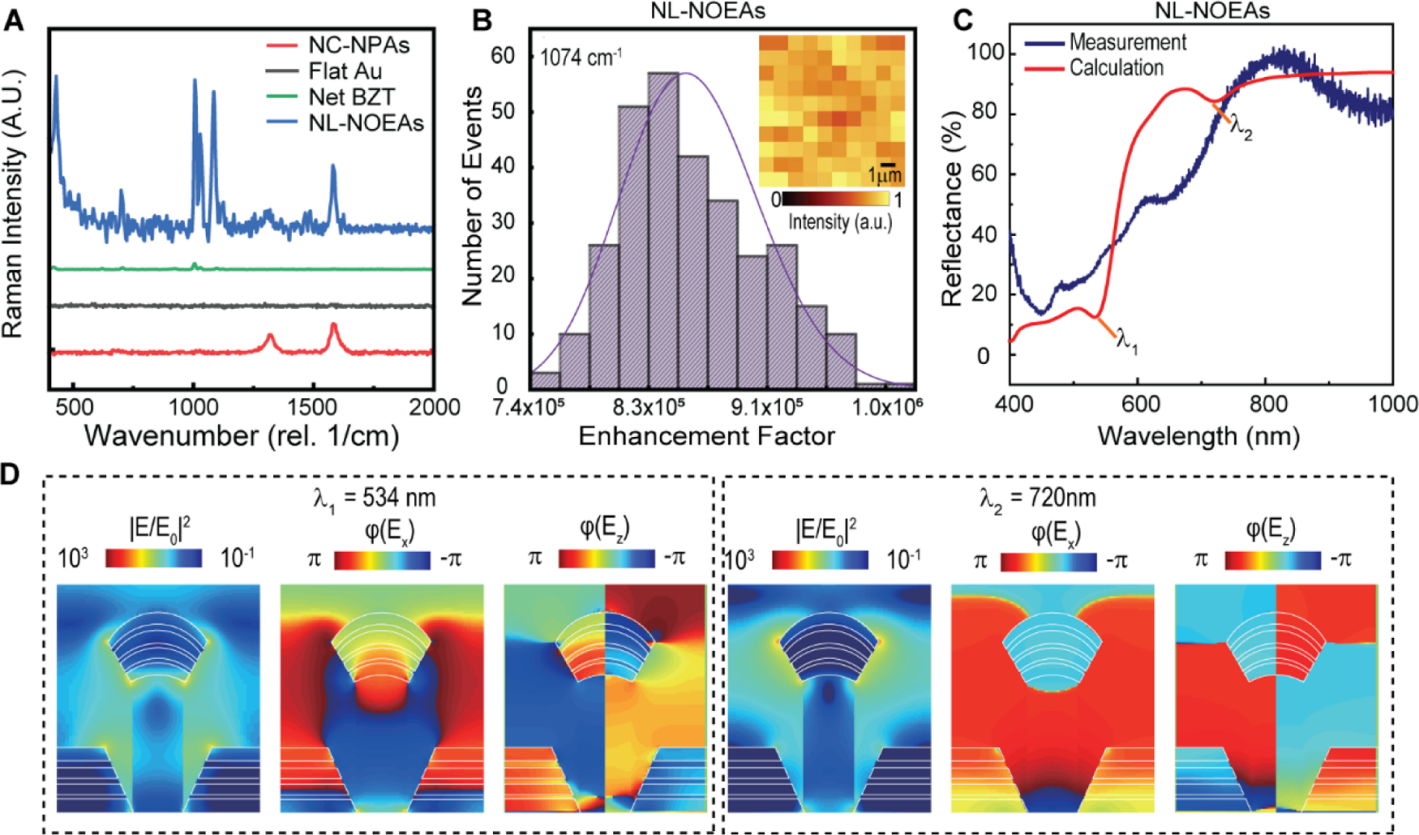
Optical properties and SERS performance of NL-NOEAs. (A) Raman spectra for four distinct samples: MWCNT/PU nanocomposite nanopillar array (NC-NPA), planar gold on flat MWCNT/PU layer (Flat Au), bare benzotriazole (Net BZT), and BZT-modified NL-NOEAs. (B) Histograms of SERS enhancement factors (EFs) for NL-NOEAs, which a 2D Raman image (inset) of a 20 μm × 20 μm area for the BZT Raman peak at 1074 cm^–1^. (C) Experimental and finite-difference time-domain (FDTD) calculated reflectance spectra of NL-NOEAs. (D) FDTD-calculated near-field distribution maps of |*E*|^2^, φ(*E*_*x*_), and φ(*E*_*z*_) in the *x*–*z* plane for NL-NOEAs at resonant wavelengths of 534 and 720 nm.

To evaluate the SERS enhancement factor (EF) of NL-NOEAs, 2D Raman measurements were conducted on benzenethiol (BZT)-functionalized samples. These measurements allowed benchmarking NL-NOEA SERS performance against control samples: bare NC-NPAs, flat Au, and neat BZT (Figure 2A,B). Figure 2A displays the average BZT Raman spectra obtained from 10 × 10 pixels over a 10 × 10 μm^2^ area using a 20× objective lens (NA ≈ 0.4) under 785 nm laser excitation (2 mW power, 0.5 s integration time per pixel). NL-NOEAs exhibited exceptional SERS performance with distinct BZT Raman features, while control samples showed no or weak BZT Raman signatures due to the absence of plasmonic enhancement. The NC-NPAs revealed two prominent Raman peaks at 1340 and 1580 cm^–1^ corresponding to the D-band and G-band of MWCNT,^43^ originating from out-of-plane vibrations due to structural defects and in-plane vibrations of sp^2^-bonded carbon atoms, respectively. Carboxyl-functionalized MWCNTs were used as filler materials to enhance the conductivity of NL-NOEAs by lowering the electrical percolation threshold.^44^

The uniformity of SERS hot spots was assessed using 2D Raman mapping across multiple positions (Figure 2B). The histogram of SERS enhancement factors (EFs) at 1074 cm^–1^ from 300 pixels over three distinct regions follows a Gaussian distribution, indicating uniformly distributed SERS hotspots. SERS EFs were determined using the following equation:^33^ EF = (*I*_SERS_/*N*_SERS_)/(*I*_Raman_/*N*_Raman_), where *I*_SERS_ and *I*_Raman_ are the BZT SERS and net BZT Raman intensities, and *N*_SERS_ and *N*_Raman_ are the number of BZT molecules contributing to each signal. The SERS EFs of NL-NOEAs ranged from ≈7.4 × 10^5^ to ≈1 × 10^6^ (Figure 2B). The minimal variations of EFs across different positions demonstrate the consistent SERS performance of the scalable NL-NOEAs, making them suitable for practical SERS and EK-SERS applications.

To investigate the optical properties of NL-NOEAs, broadband microreflectance spectroscopy and three-dimensional (3D) finite-difference time-domain (FDTD) simulations (Lumerical Inc.) were performed (Figure 2C). The FDTD calculations, informed by FIB-SEM images, employed structural and material models consisting of cone-shaped Au and Ag layers on the CNT nanopillar and planar Au and Ag layers on the substrate. The incident plane wave was perpendicular to the substrate along the *z*-direction, with electric fields aligned along the *x*-direction. The refractive index of the MWCNT-doped PU, obtained from ellipsometry measurements on a planar sample, was included in the model. As shown in Figure 2C, the measured reflectance spectrum shows low reflectance below 500 nm due to gold interband transitions. Within 500–1000 nm, measured reflectance dips occurred at 660 and 986 nm, while simulated dips appeared at 534 and 720 nm (Figure 2C).

The discrepancy between the measured and simulated spectra can be attributed to several factors inherent to the complexity of the NL-NOEA structure and the challenges in precisely replicating its geometry and material properties in the FDTD model. Subtle variations in nanocavity shapes, metal surface roughness arising from the deposition process, the angled component of incident light in microreflectance measurements, and the inherent nonuniformity of the MWCNT/NOA83H nanopillar array can all contribute to the observed differences. These factors lead to inhomogeneous broadening and potential shifts in the reflectance features. It is important to note that while the experimental reflectance near 785 nm is high, suggesting limited average plasmonic enhancement, this does not negate the observed SERS enhancement. SERS is highly localized at plasmonic hotspots, and even a small fraction of well-defined hotspots can lead to substantial SERS enhancement.

Figure 2D presents near-field simulations at 534 and 720 nm, revealing two distinct plasmonic modes with in-phase φ(***E***_***x***_) distributions characteristic of electric dipole (ED) modes, concentrating |***E***|^2^ at the edges of nanolaminate nanoparticles atop nanopillars. The 534 nm feature arises from hybridization between the off-resonant plasmonic ED mode of nanolaminate nanoparticles and delocalized Bloch SPP modes supported by nanohole arrays. The 720 nm feature stems from the resonant ED mode of nanolaminate nanoparticles. Examining these modes allows for deeper understanding of light-matter interactions within NL-NOEAs, enabling tailored optical properties and design optimization.

**Figure 3:**
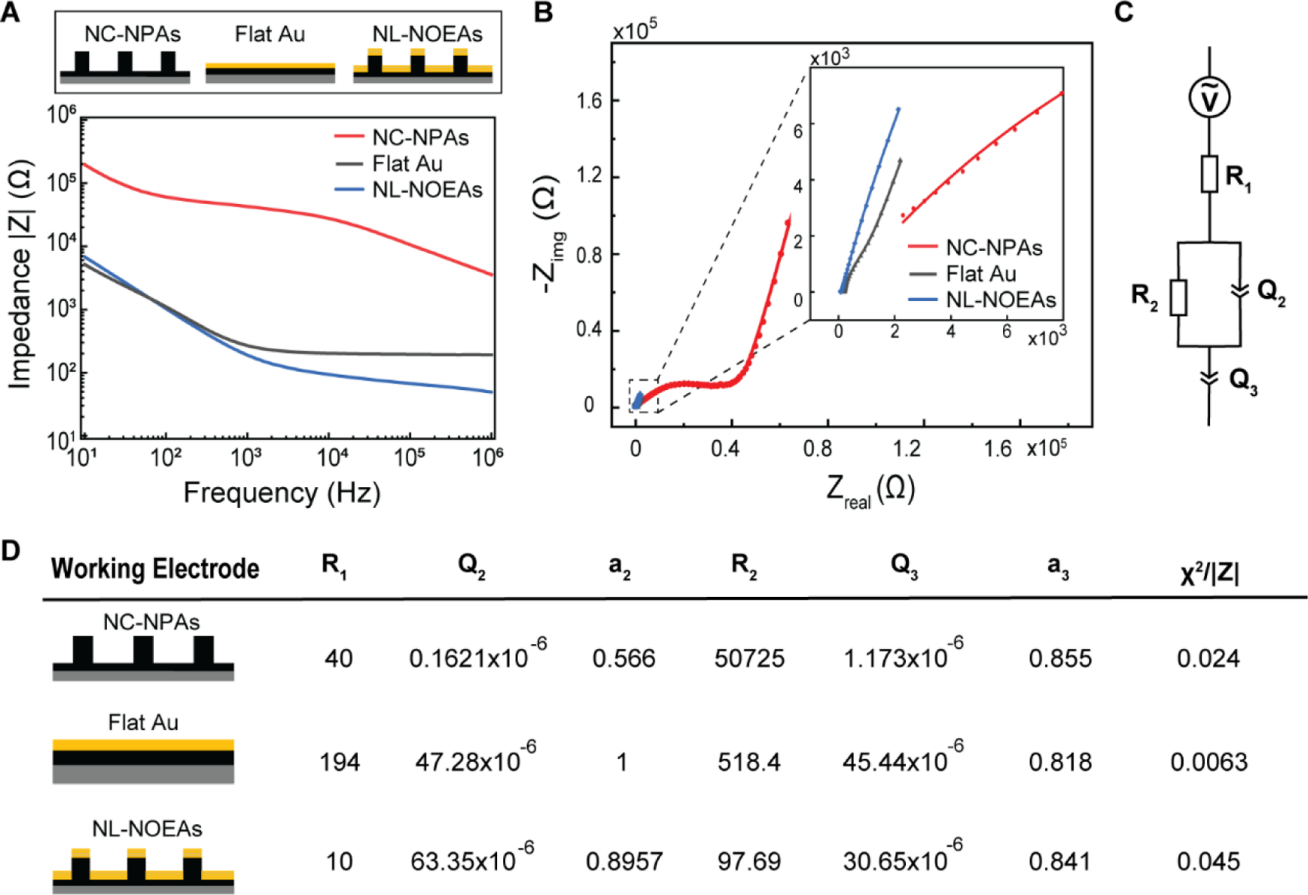
Impedance characterization and modeling of NL-NOEAs in an ionic solution. (A) Measured impedance magnitude |Z| plots and (B) Nyquist plots for MWCNT/PU NC-NPA, planar gold (Au) on flat MWCNT/PU layer (Flat Au), and NL-NOEAs in 1× phosphate-buffered saline (PBS) solution, with circuit model-fitted curves. (C) Equivalent circuit model. (D) Table of fitted parameters for the model, allowing quantitative comparison of impedance properties between the three sample types.

To investigate the influence of NL-NOEAs’ interfacial properties on their EK-SERS performance, we employed electrochemical impedance spectroscopy (EIS) to characterize the electrical double layer (EDL) capacitance and ion diffusion behavior at the electrode–electrolyte interface (Figure 3). Measurements were conducted in a physiologically relevant 1× phosphate buffer saline (PBS) solution (pH ≈ 7.4) using a custom electrochemical cell, comparing NL-NOEAs to control samples (bare NC-NPAs and a flat Au electrode). Figure 3A demonstrates that the deposition of plasmonic metal layers for both the flat Au electrode and NL-NOEAs led to an order-of-magnitude reduction in impedance magnitude (|Z|) compared to NC-NPAs across a frequency range of 10 Hz to 100 kHz. This decrease is attributed to the superior conductivity of the metal layer over the MWCNT/PU composite. The increased surface area of NL-NOEAs further reduced |Z| compared to the flat Au electrode in the 80 Hz to 100 kHz range. At 1 kHz, relevant for electrophysiological measurements, |Z| decreased from 201.7 Ω (flat Au) to 90.2 Ω (NL-NOEAs). To assess the electrochemical performance of NL-NOEAs as an EK-SERS substrate, we analyzed the Nyquist plot and developed an equivalent circuit model (Figure 3B,C). The Nyquist plot, representing resistive (real part) and capacitive reactance (negative imaginary part) characteristics, enabled us to construct the equivalent circuit for each sample. Figure 3B displays experimental data (dots) fitted with the model (lines). The addition of the plasmonic metal layer significantly reduced impedance, evident in both the real and imaginary parts. The larger surface area of NL-NOEAs resulted in higher EDL capacitance compared to the planar electrode, reflected in the larger imaginary part amplitude in the Nyquist plot despite similar real parts.

Considering the microscopic distribution of electrons and ions at the electrode–electrolyte interface, we constructed a model (*R*_1_ + *Q*_2_//*R*_2_ + *Q*_3_) to fit the Nyquist plot for the different samples (Figure 3C). In this model, R_1_ represents bulk ohmic resistance, *Q*_2_ and *R*_2_ are the double-layer capacitance and the charge transfer resistance at the electrode–electrolyte interface, respectively, and *Q*_3_ denotes the anomalous ion diffusion impedance from the electrode to the electrolyte. The fitted component values and fitting indicator parameter (χ^2^/|Z|) are provided in Figure 3D. *R*_1_ comprises three series resistances: electrolyte resistance (*R*_1_′), MWCNT/PU nanocomposite resistance (*R*_1_″), and parasitic contact and equipment resistance (*R*_1_‴). The negligible *R*_1_ value only causes a small horizontal shift in the Nyquist plot. *Q*_2_ signifies the EDL capacitance, which is smaller for NC-NPAs than NL-NOEAs and flat Au due to the significantly increased surface area at the Au-electrolyte interface.

The *a*_2_ term (0 ≤ *a*_2_ ≤ 1) characterizes the constant phase element (CPE), ranging from an ideal capacitor (*a*_2_ = 1) to an ideal resistor (*a*_2_ = 0). The larger value (*a*_2_ ≈ 0.8) for NL-NOEAs suggests their more capacitor-like behavior, attributed to the relatively smooth Au surface. In contrast, the smaller value (*a*_2_ ≈ 0.566) for NC-NPAs indicates significant deviation from ideal capacitor due to their rough and heterogeneous MWCNT/PU surfaces. *R*_2_ represents charge transfer resistance, which is larger for NC-NPAs due to charge transfer through the MWCNT layer. NL-NOEAs have a smaller *R*_2_ than flat Au due to increased surface area, facilitating charge transfer. The *Q*_3_ term accounts for anomalous ion diffusion impedance, relating to the electrode–electrolyte interface structure. The similar *Q*_3_ values across all samples suggest that the difference in microscopic electrode surface roughness is negligible compared to the ion diffusion distance in the electrolyte. The fitting indicator parameter χ^2^/|Z| assesses the model’s accuracy, with smaller values indicating *a* better fit. The relatively small χ^2^/|Z| values for all three sample types confirm that our equivalent circuit model accurately fits the measured Nyquist plots in Figure 3D, validating its applicability for understanding the electrochemical behavior of the different samples.

To investigate the effect of EK modulation on the SERS response of charged molecules at plasmonic hotspots, we used NL-NOEAs to perform DC EK-SERS measurements on 10^–5^ mol/L R6G in 1× PBS. R6G, a positively charged molecule (+1*e* at pH ≈ 7.4), was chosen as a model analyte due to its charge-dependent adsorption behavior on the Au electrode surface. The use of 1× PBS as the electrolyte serves the dual purpose of mimicking a physiologically relevant environment and providing sufficient ionic strength to support electrokinetic phenomena. While the high ionic strength in the EDL may influence the polarizability of R6G and its Raman scattering efficiency through ion pairing and local field effects, the primary focus of this study is to demonstrate the effectiveness of EK-SERS in modulating the concentration and orientation of R6G at the electrode surface.

**Figure 4:**
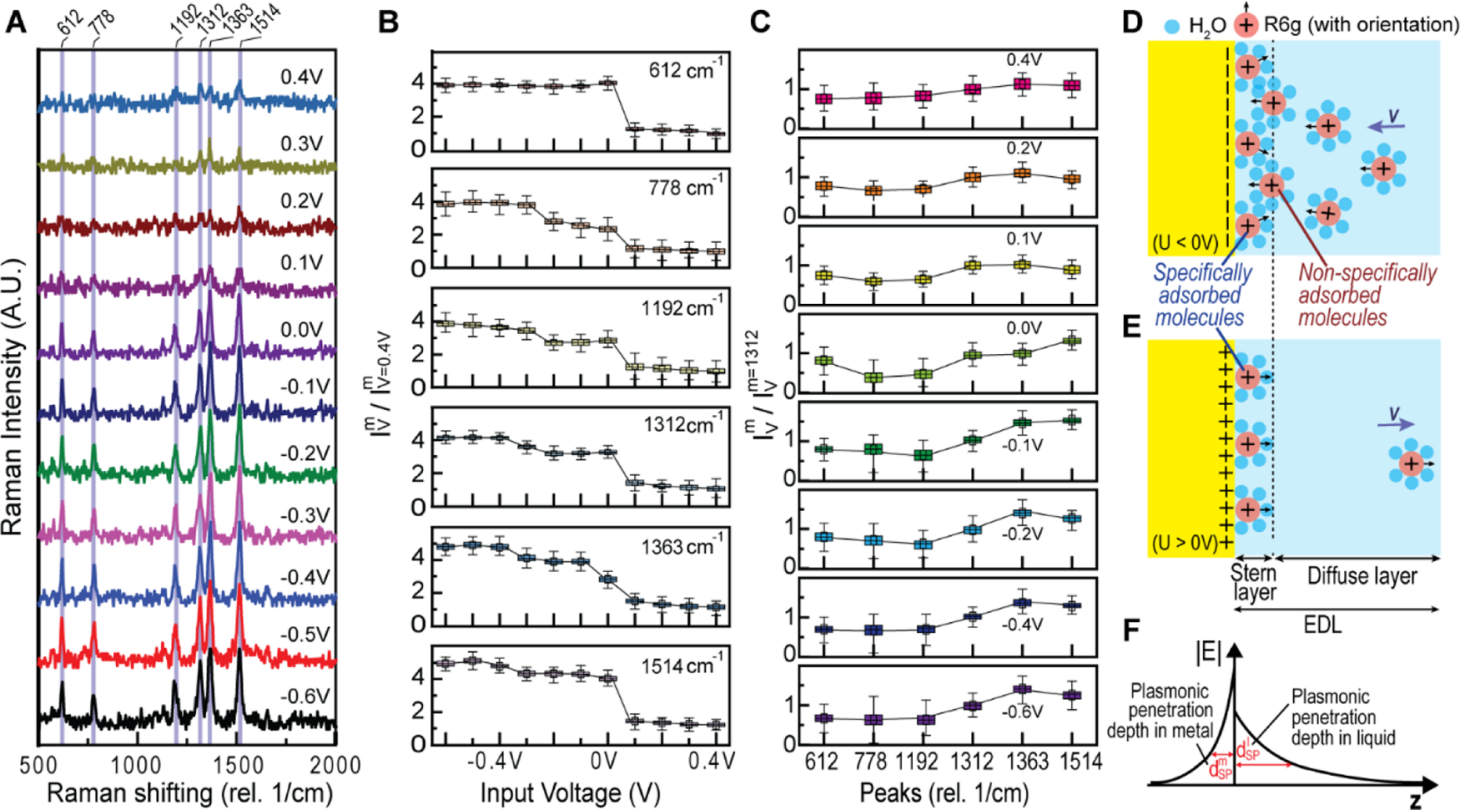
DC EK-SERS measurements of charged analyte molecules using NL-NOEAs. (A) Averaged Raman spectra of 10^–5^ mol/L R6G in 1× PBS for 100 s measurements with 1 s integration time under DC voltage input from −0.6 to 0.4 V (0.1 V increments), offset on the *y*-axis for clarity. (B) Box plot of six featured R6G SERS peak intensity ratios (*I*_*v*_^*m*^/*I*_*v* = 0.4*V*_^*m*^) vs input voltage, where *m* represents vibration modes (peaks), and *v* denotes input voltage. Each R6G peak intensity is normalized to its minimum value at *V* = 0.4 V. Boxes represent 100 data points, with top, middle, and bottom bars indicating max, median, and min values, respectively, and the central square showing the average value. (C) Box plot of six featured R6G SERS peak intensity ratios (*I*_*v*_^*m*^/*I*_*v*_^*m* = 1312^) normalized to the intensity of peak 1312 cm^–1^ at the same voltage. (D, E) Illustrations of the local environment at the electrode–electrolyte interface with (D) positive and (E) negative electrode potential. (F) Schematic of plasmonic enhancement intensity |E|^4^ profile at the electrode–electrolyte interface.

As shown in Figure 4A, the average SERS spectra collected under constant DC voltage inputs ranging from −0.6 to 0.4 V (with 0.1 V increments) revealed a voltage-dependent trend in R6G peak intensities. The maximum peak intensity was observed at −0.6 V, corresponding to electrostatic attraction of the positively charged R6G to the negatively charged electrode surface. Conversely, the minimum peak intensity occurred at 0.4 V, due to electrostatic repulsion. However, even at 0.4 V, some R6G molecules remained in the hotspot region, indicating the presence of specifically adsorbed R6G within the Stern layer. This observation suggests that, in addition to electrostatic interactions, other forces contribute to R6G adsorption. We attribute this persistent signal under positive bias to the hydrophobic nature of the xanthene core in the R6G molecule. The hydrophobic xanthene core is driven toward the Au electrode surface even under positive bias due to favorable hydrophobic interactions, leading to strong adhesion that resists electrostatic repulsion. This interplay between electrostatic forces and hydrophobic interactions results in the observed robust R6G signal across the entire voltage range.

To further analyze the data, we plotted the peak intensity ratio (normalized to the minimum intensity at 0.4 V) against the applied voltage for six prominent Raman peaks associated with R6G (Figure 4B). This analysis revealed that the concentration of R6G molecules in the plasmonic hotspots decreased with increasing electrode potential (eqs 1–4). In the positive voltage range (0.1 to 0.4 V), the SERS intensities of all peaks exhibited minimal change, suggesting that free-moving R6G molecules (including nonspecifically adsorbed and volumetric molecules) were repelled from the hotspot, while the concentration of specifically adsorbed R6G remained relatively constant.

In the negative voltage range (−0.1 to −0.6 V), the SERS intensities of most peaks increased, indicating an increase in R6G concentration at the electrode surface. The 612 cm^–1^ peak, attributed to the C–C–C ring in-plane bending mode, which is insensitive to molecular orientation changes upon electrode potential modulation, served as a concentration indicator, revealing R6G concentration saturation under negative voltage input. Changes in other peaks within this range were attributed to differences in R6G orientation, with a distinct pattern change observed between −0.1 to −0.3 and −0.4 to −0.6 V. This suggests that R6G adopts different orientation configurations as the input DC E-field intensities vary, resulting in altered Raman cross sections. The significant increase in peak intensity ratio observed during the transition from positive to negative voltage, particularly between 0.1 and −0.1 V, is attributed to the DC E-field polarity flip, which significantly alters the concentration of free-moving R6G in the hotspot.

To isolate the effect of molecular orientation from concentration effects, we plotted the ratio of the same six peaks to the 1312 cm^–1^ peak at the same voltage (Figure 4C). This analysis revealed changes in the R6G peak index-dependent ratio pattern as the electrode voltage transitioned from positive to negative (0.1 to −0.1 V), suggesting an alteration in R6G orientation under the opposite DC E-field direction (eqs 5 and 6). The pattern at 0 V, obtained before applying any voltage, served as a reference for the neutral state orientation. The deviation of the ratio pattern at 0.1 V from those at 0 and −0.1 V highlights the sensitivity of R6G orientation to the applied voltage. Specifically, the ratio of peaks 1363 and 1514 cm^–1^ to peak 1312 cm^–1^ was larger at 0 V than at 0.1 V, and even larger at −0.1 V. Conversely, the ratio of peaks 778 and 1192 cm^–1^ to peak 1312 cm^–1^ decreased as the voltage shifted from 0.1 to −0.1 V.

In the negative voltage input range (−0.1 to −0.6 V), a consistent R6G peak index-dependent ratio pattern was observed for peaks at 612, 778, and 1192 cm^–1^ (Figure 4C). However, the ratio of peak 1363 to 1312 cm^–1^ increased, while the ratio of peak 1514 cm^–1^ to peak 1312 cm^–1^ decreased with increasing negative voltage. This observation suggests that R6G adopts different orientation configurations as the input DC E-field intensities vary, with the orientation change primarily dominated by the volumetric R6G, as the concentration of the specifically adsorbed R6G is saturated. In the positive voltage range (0.1 to 0.4 V), specifically adsorbed R6G molecules predominantly contribute to the measured SERS spectra. Subtle differences in peak index-dependent ratio patterns were observed, with the ratio of peaks 778 and 1192 cm^–1^ to peak 1312 cm^–1^ increasing as voltage became more positive. This change is attributed to the orientation of specifically adsorbed R6G, as free-moving R6G molecules are repelled from the hotspot before the measurement.

Figure 4D schematically represents the distribution of R6G molecules at the Au-electrolyte interface under negative (<0 V) and positive (>0 V) voltage conditions. Under negative voltage, positively charged R6G molecules migrate and accumulate at the negatively charged electrode surface. Free-moving R6G molecules (including nonspecifically adsorbed and volumetric molecules) can traverse in and out of hotspots with voltage input (Figure 4D) and exhibit changes in their orientation in response to the electric field direction. The orientation of surface-bound R6G molecules also changes, albeit to a lesser extent, than free-moving R6G molecules in the electrolyte. Notably, free-moving R6G molecules dominate the measured SERS spectra for the negative voltage range (−0.1 to −0.6 V) over specifically adsorbed molecules.

The SERS measurement captures the R6G signal under a specific voltage at equilibrium. The surface plasmon-localized |***E***|-field decays exponentially with distance *z* from the metal–electrolyte interface (Figure 4F), enhancing the Raman signal by a factor of |***E***|^4^ for all R6G molecules within the hotspots. The resulting SERS spectra combine signals from specifically and nonspecifically adsorbed R6G, as well as volumetric R6G molecules, within plasmonic hotspots. Overall, in solutions with physiological ionic strength (∼150 mMol/L), where the electrolyte Debye length *d*_D_^*l*^ is ≈0.79 nm, the interplay between electrostatic forces and other interfacial forces (e.g., hydrophobic interactions, van der Waals electrode-R6G forces, etc.) governs R6G adsorption and orientation at the electrode surface. This balance allows for modulation of analyte adsorption and orientation by tuning the electrode potential, producing voltage-dependent SERS fingerprints and enabling high-order EK-SERS.

**Figure 5:**
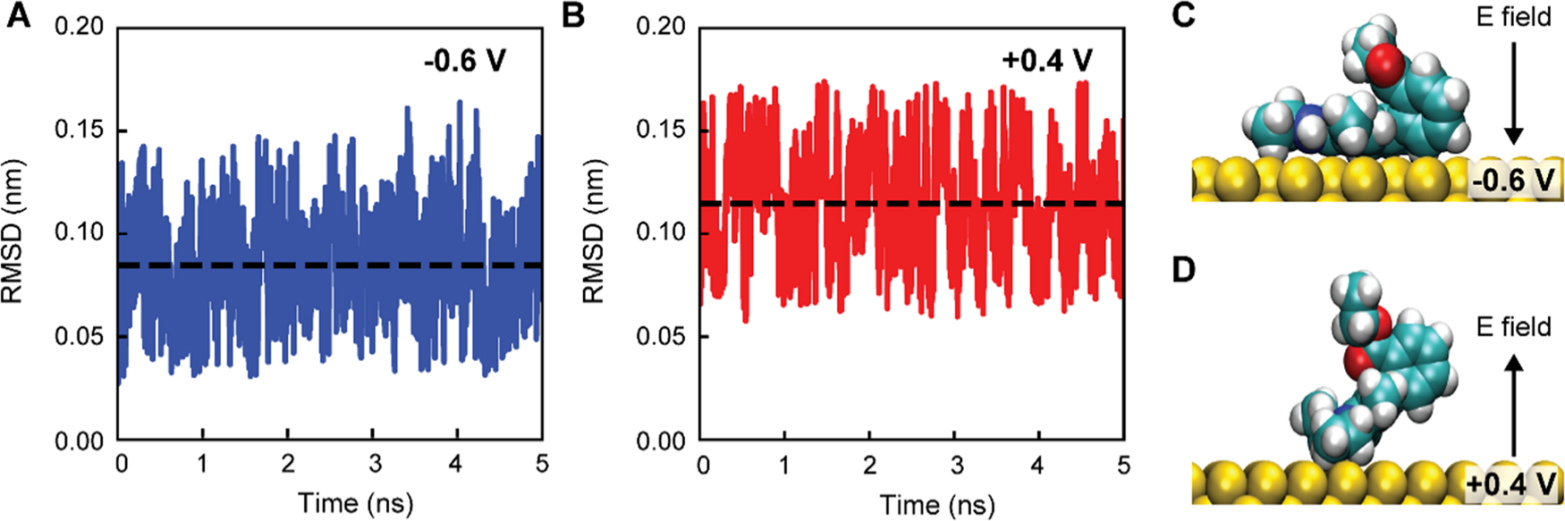
Molecular dynamics simulations of R6G adsorption on gold electrodes under different voltages. (A, B) Root mean square deviations (RSMD) of an R6g molecule adsorbed on gold electrodes with a voltage of −0.6 V (A) and +0.4 V (B), with the structure at −0.6 V and 100 ns taken as the reference. The black dashed line shows the mean RMSD value within 5 ns. (C, D) Snapshots of R6G molecules adsorbed on gold electrodes with a voltage of −0.6 V (C) and +0.4 V (D). Water molecules and ions are omitted for clarity.

To further elucidate the voltage-dependent adsorption behavior of R6G molecules observed in the EK-SERS experiments (Figure 4), molecular dynamics (MD) simulations were performed. The evolution of the root-mean-square deviation (RMSD) for an R6G molecule adsorbed on a negatively charged gold electrode (−0.6 V) is shown in Figure 5A, using its structure at 100 ns as a reference. The average RMSD is ∼0.08 nm with a variance of ∼5.4 × 10^–4^ nm^2^. In contrast, the RMSD for a specifically adsorbed R6G molecule on a positively charged gold electrode (+0.4 V) exhibits a higher mean value (∼0.11 nm) with a comparable variance of ∼6.7 × 10^–4^ nm^2^ (Figure 5B).

The distinct adsorption configurations of R6G on differently charged electrodes, as observed in Figure 5A,B, are illustrated in Figure 5C,D. On the negatively charged electrode, the R6G molecule adsorbs its xanthene ring system parallel to the electrode surface (Figure 5C). Conversely, on the positively charged electrode, the R6G molecule is anchored through its ethyl groups, causing the xanthene ring system to tilt at an angle relative to the electrode (Figure 5D). This difference in adsorption configuration is likely due to the electrostatic repulsion between the positively charged xanthene ring of the R6G molecule and the positively charged electrode. These MD simulations provide further evidence that the applied voltage can modulate the adsorption configuration of R6G molecules on the electrode surface, which in turn affects their SERS response.^34^ The observed changes in molecular orientation and structure under different voltage conditions are consistent with the voltage-dependent SERS peak intensity ratios observed in the experimental results (Figure 4), highlighting the potential of EK-SERS for probing the dynamic behavior of molecules at the electrode–electrolyte interface.

**Figure 6:**
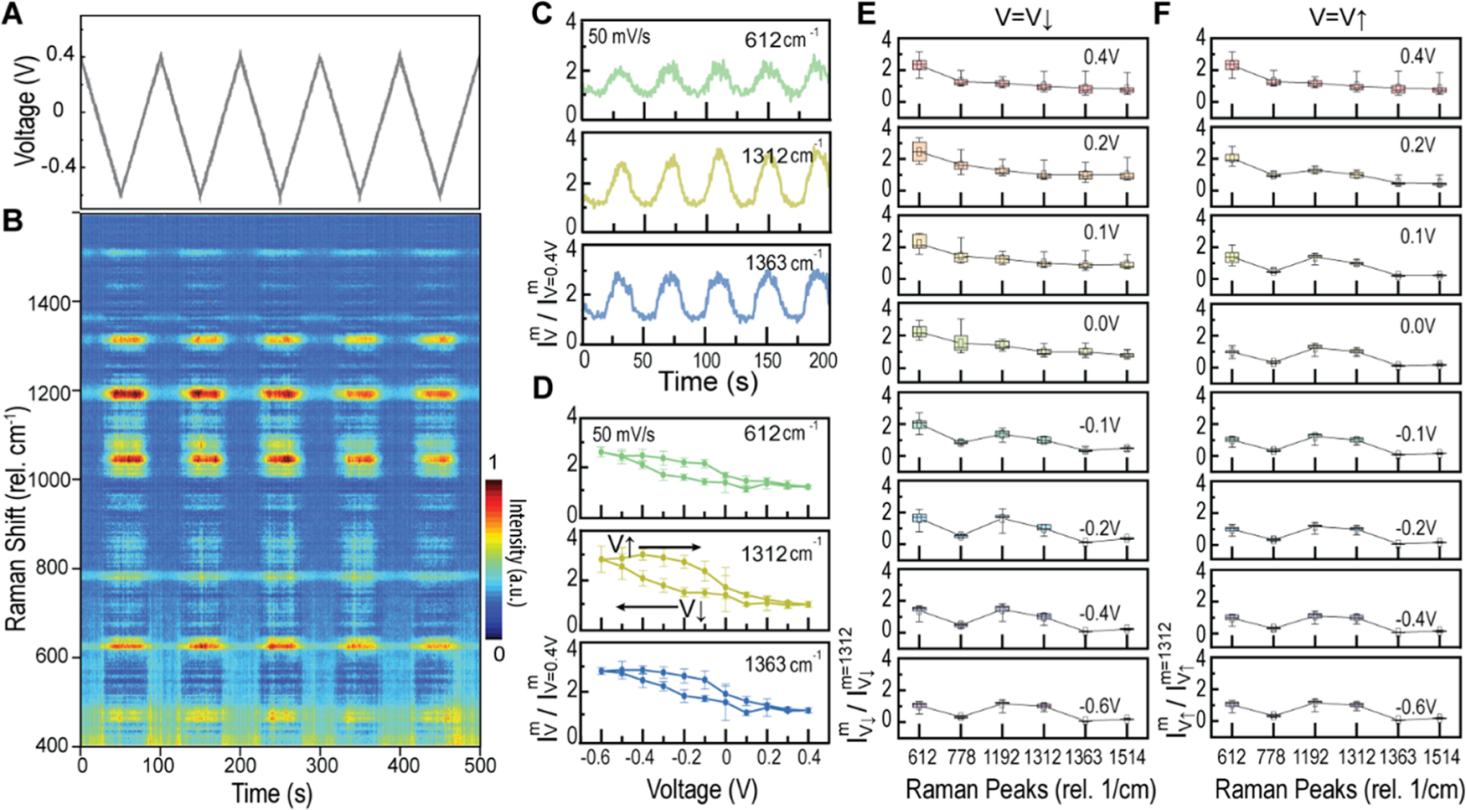
AC EK-SERS measurements of charged analyte molecules using NL-NOEAs. (A) Time-dependent voltage input under cyclic voltammetry (CV) sweeping between −0.6 and 0.4 V. (B) Time-dependent SERS spectra of 10^–5^ mol/L R6G from NL-NOEAs sample in 1× PBS under 785 nm laser excitation. (C) Time-dependent SERS peak intensity ratios (*I*_*v*_^*m*^/*I*_*v* = 0.4*V*_^*m*^) for peaks at 612, 1312, and 1363 cm^–1^, normalized to their minimum values at *V* = 0.4 V. (D) SERS peak ratio plot for five cycles of peaks at 612, 1312, and 1363 cm^–1^ at different input voltages during sweep down (V↓, upper trace) and sweep up (V↑, lower trace), with standard deviation as error bars, normalized to their minimum values at V = 0.4 V. (E, F) SERS peak intensity ratio (*I*_*v*_^*m*^*/I*_*v*_^*m* = 1312^) box plots for five cycles of six feature peaks at different voltages during (E) sweep down (V↓) and (F) sweep up (V↑), with peak intensities normalized to peak 1312 cm^–1^ at the same voltage.

To gain deeper insights into the dynamic behavior of charged molecules under AC electrokinetic (EK) modulation, we extended our investigation beyond DC EK-SERS and performed AC EK-SERS measurements on 10^–5^ Mol/L Rhodamine 6G (R6G) in 1× PBS using NL-NOEAs (Figure 6). This approach allows us to probe molecular transport processes and analyte behavior under nonequilibrium conditions, complementing the steady-state information obtained from DC EK-SERS.

Figure 6A,B shows the applied AC voltage waveform for EK modulation and the time-dependent SERS spectra of R6G under 785 nm laser excitation, respectively. The AC EK-SERS maps reveal a stronger overall Raman peak intensity under negative voltage input, consistent with the attraction of positively charged R6G molecules to the negatively charged electrode. Conversely, peak intensity is lower under positive voltage due to electrostatic repulsion. However, specifically adsorbed R6G molecules remain on the electrode surface even at positive voltages due to their hydrophobicity. The emergence of new peaks around 1050 cm^–1^ suggests a distinct R6G orientation during dynamic motion compared to the static state under DC input. This highlights the potential of AC EK-SERS for high-order multidimensional analysis of charged analytes in ionic solutions, where Raman fingerprint data, AC input range, frequency, and sweep direction can be considered.

To further analyze AC EK-SERS dynamics, we plotted the intensity ratio of peaks at 612, 1312, and 1363 cm^–1^, normalized to their minimum values at *V* = 0.4 V, as a function of time (Figure 6C). The varying scale of ratio changes among peaks reflects the combined contributions of specifically adsorbed and free-moving R6G, as well as their orientation and concentration changes at the plasmonic hotspots. In the positive voltage range (0.1 to 0.4 V), minimal changes in the R6G peak ratios suggest that most free-moving R6G is repelled from the hotspot, leaving specifically adsorbed R6G as the dominant contributor. Conversely, significant changes in the R6G peak ratios occur in the negative voltage range (−0.1 to −0.6 V), primarily due to variations in R6G concentration at the hotspot. The attraction of positively charged R6G molecules under negative voltage leads to a large influx of volumetric R6G, dominating the SERS signal.

Figure 6D depicts the average peak intensity ratio (*I*_*m*_^*V*^/*I*_*m*_^*V*=0.4^) of peaks at 612, 1312, and 1363 cm^–1^ at different voltages for five cycles, with the top trace corresponding to voltage increasing from −0.6 to 0.4 V and the bottom trace representing voltage decreasing from 0.4 to −0.6 V. All peaks exhibit a hysteresis pattern, serving as an R6G fingerprint. This hysteresis, particularly prominent in the negative voltage range, likely arises from the difference between the diffusion speed of R6G from the bulk to the EDL interface and the faster electromigration-driven drift speed of R6G toward the hotspot.

As the AC voltage sweeps from −0.1 to −0.6 V, the continuous increase in R6G peak intensity indicates a constant increase in R6G concentration at the hotspot. However, the diffusion of R6G from the bulk is slower than the EK-driven drift, resulting in an insufficient amount of R6G at the interface and limiting the attraction process. The SERS signal remains dominated by free-moving R6G. When the AC sweeps from −0.6 to −0.4 V, the R6G peak ratio increases, particularly for the 1312 cm^–1^ peak, due to the stronger drifting force from the AC E-field overcoming the concentration gradient-driven drift, leading to continuous R6G accumulation at the hotspot. As the AC sweeps from −0.4 to −0.1 V, the R6G peak ratio decreases with increasing voltage, as the diminishing drifting force from the AC E-field allows the concentration gradient-driven drift to repel R6G back into the bulk. However, the hysteresis behavior arises from the influence of the previous state on R6G concentration.

In the positive voltage range, minimal hysteresis is observed. The E-field-driven desorption process is rapid, and as the voltage changes from 0 to 0.1 V, most free-moving R6G molecules are repelled from the hotspots, leaving specifically adsorbed R6G as the dominant contributor to the SERS signal. This suggests that the R6G signal in this range is mainly determined by the orientation of specifically adsorbed R6G, with minimal contribution from concentration changes (eqs 1–4).

The ratio of peak intensities relative to 1312 cm^–1^ during voltage sweeping down (0.4 to −0.6 V) and up (−0.6 to 0.4 V) is shown in Figure 6E,F, respectively. During the downward sweep, specifically adsorbed R6G dominates in the positive range (0.4 to 0.1 V), as volumetric R6G is rapidly repelled. The ratio pattern, primarily reflecting orientation information, remains similar in this range, indicating minimal changes in R6G orientation. A noticeable change in the pattern occurs in the transition region (0.1 to −0.1 V), with the ratio of peaks at 778 and 1363 cm^–1^ decreasing from 0 to −0.1 V. This change, different from that observed under DC input, is attributed to differences in the E-field and R6G density between AC and DC conditions. In the negative range (−0.1 to −0.6 V), the R6G orientation slowly changes, with peak 1192 cm^–1^ slightly increasing from −0.1 to −0.4 V and then decreasing from −0.4 to −0.6 V, while peak 1363 cm^–1^ consistently decreases. This orientation change is likely dominated by volumetric R6G, as more molecules are drawn into the hotspot, while the orientation of specifically adsorbed R6G remains relatively stable.

During the upward sweep (Figure 6F), the R6G orientation is more stable in the negative range (−0.1 to −0.6 V), with an almost constant ratio pattern across all voltages. This supports the hypothesis that the variation observed during the downward sweep is due to the need for volumetric R6G to align with the E-field direction from its previous state. In the transition region (−0.1 to 0.1 V), the 0 V pattern slowly evolves, with the ratio of peaks at 778 and 1363 cm^–1^ increasing as the voltage increases. This is again attributed to the difference in the E-field and R6G density between AC and DC conditions. In the positive range (0.1 to 0.4 V), the pattern continues to change, with the ratio of peaks at 778 and 1363 cm^–1^ still increasing. This change is dominated by specifically adsorbed R6G, as free-moving R6G has already been repelled. The subtle change in the ratio pattern suggests that the orientation of specifically adsorbed R6G can be modified by the E-field (eqs 5 and 6).

**Figure 7:**
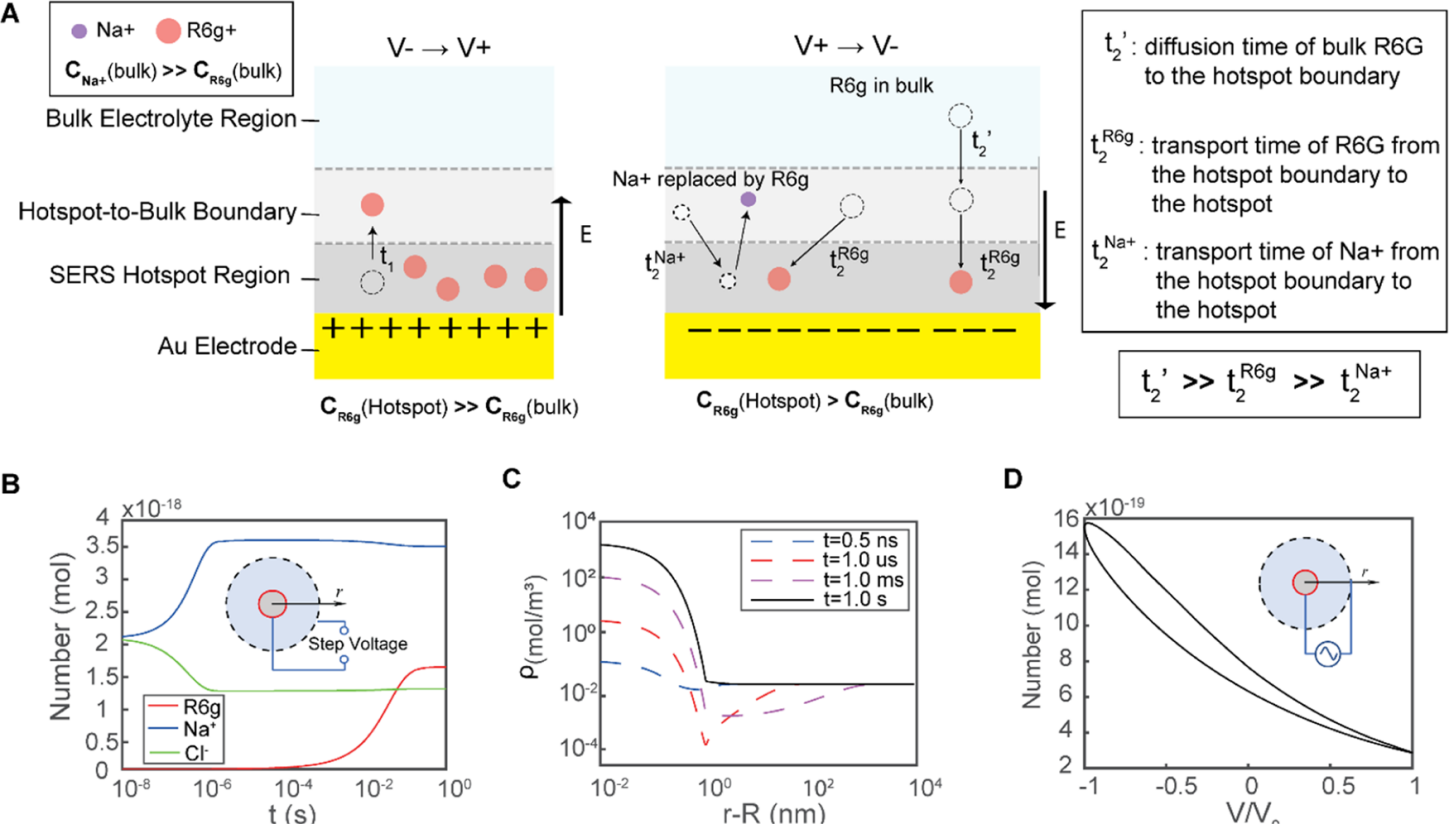
Numerical simulation of R6G and ions in response to DC and AC voltages. (A) Schematic of the simulation setting. (B, C) Ions and R6G dynamics near a spherical electrode impulsively switched to −75 mV relative to the bulk. (B) The evolution of adsorbed Na^+^, Cl^–^, and R6G^+^ in the interfacial zone (1 nm from the electrode). (C) R6G concentration profiles along the solution domain at different time. (D) The cyclic evolution of adsorbed R6G amount with an applied 1 Hz AC voltage to the electrode (data are shown at the periodic steady state).

To elucidate the mechanistic origin of the large time scale of R6G transport observed in the AC EK-SERS experiments (Figure 6), we conducted numerical simulations to investigate the dynamics of ions and R6G molecules near the electrode surface under the influence of applied DC and AC voltages. The simulation setup consisted of a spherical electrode immersed in a 1× PBS solution containing 10^–5^ Mol/L R6G. The spatiotemporal evolution (Figure 7A) of ion concentrations under spherical coordinates are governed by the Nernst–Planck equation and the Poisson equation. Upon application of a negative DC voltage (−75 mV) to the electrode, an electric field is established, generating electrostatic forces that drive the migration of ions and charged R6G molecules (the second term on the left-hand side of eqs 7–9 in Methods).

The spatiotemporal evolution of ion and R6G concentrations near the electrode surface is shown in Figure 7B,C. Initially (0–4 μs), Na^+^ ions rapidly accumulate near the electrode, while Cl- ions are depleted, forming an electrical double layer (EDL) within the nanometer scale. This EDL effectively screens the electric field in the bulk solution away from the electrode. Although R6G molecules are favored near the electrode due to nonelectrostatic forces (the third term on the left-hand side of eq 9), their accumulation is initially limited by their low concentration. However, after the initial rapid ion redistribution, R6G molecules continue to be driven toward the electrode due to favorable thermodynamics, leading to a gradual increase in their concentration near the surface over a longer time scale (milliseconds). This slow accumulation is attributed to the low bulk concentration of R6G and the formation of a transient depletion zone outside the range of interfacial forces, which limits the diffusion of R6G molecules toward the electrode (the first term on the left-hand side of eq 9).

Under AC voltage modulation, the voltage on the electrode periodically switches between negative and positive values. The R6G response under negative voltage qualitatively follows the same pattern as in the DC case. However, when the voltage switches to positive, the absence of a depletion zone allows for faster equilibration of R6G concentration due to the direct transport of accumulated R6G molecules from the electrode surface toward the bulk solution. This mismatch in the response times of R6G to positive and negative voltage changes results in the hysteresis observed in the AC EK-SERS measurements at low frequencies (Figure 7D), consistent with our experimental observations. The degree of hysteresis depends on both the R6G diffusivity and the AC frequency.

## Conclusions

In conclusion, our study elucidates the intricate interplay between applied voltage, R6G molecule orientation, and the resulting SERS spectral signatures. The combination of experimental observations and molecular dynamics simulations provides valuable insights into the fundamental mechanisms governing the adsorption and orientation of R6G molecules at the Au-electrolyte interface. This knowledge is crucial for understanding the SERS phenomena in a broader context and offers potential opportunities for developing advanced SERS sensor technologies. The ability to control analyte adsorption and orientation via electrode potential modulation, without influencing their chemical identity, empowers the generation of high-order EK-SERS signals, promising transformative advancements in fields such as chemical sensing, environmental monitoring, and biomedical diagnostics.

Furthermore, our comparative investigation of AC and DC EK-SERS has yielded valuable insights into the dynamic behavior of R6G molecules under varying voltage conditions. The observed differences in peak ratio patterns, orientation changes, and R6G density highlight the system’s complexities and contribute to a more nuanced understanding of the factors governing R6G-electric field interactions and their impact on SERS signals. These findings can guide future experimental designs and optimize the EK-SERS detection of R6G and other analytes.

The NL-NOEA platform, distinguished by its high SERS sensitivity and electrokinetic control, holds immense potential for revolutionizing chemical sensing. The ability to manipulate molecular behavior at the electrode–electrolyte interface through applied electric fields opens doors to unprecedented selectivity and sensitivity in analyte detection. The inherent scalability of NL-NOEAs further amplifies their practicality for real-world sensing applications.

However, challenges such as surface fouling and the need for further optimization of the fabrication process and EK modulation parameters need to be addressed to fully realize the potential of NL-NOEAs in real-world sensing devices. Future research efforts should focus on developing effective strategies to mitigate fouling, potentially through EK-driven surface regeneration and the use of antifouling coatings. Additionally, exploring hybrid micronanofabrication approaches and integrating functional materials could enhance the scalability and performance of NL-NOEAs, expanding their applicability in diverse sensing scenarios. By overcoming these hurdles, we envision the realization of practical and robust EK-SERS sensors based on NL-NOEAs. These sensors, capable of generating high-order EK-SERS signals, have the potential to reshape chemical sensing across diverse fields, driving advancements in environmental monitoring, food safety, healthcare diagnostics, and beyond.
